# Effect of dolutegravir-based first-line antiretroviral therapy on weight and body mass index among adult people living with HIV on follow up at health facilities in Hawassa city administration, Southern Ethiopia: a retrospective cohort study

**DOI:** 10.1080/07853890.2023.2242250

**Published:** 2023-08-02

**Authors:** Agete Tadewos Hirigo, Daniel Yilma, Ayalew Astatkie, Zelalem Debebe

**Affiliations:** a School of Medical Laboratory Science, College of Medicine Health Sciences, Hawassa University, Hawassa, Ethiopia; b Center for Food Science and Nutrition, Addis Ababa University, Addis Ababa, Ethiopia; c Department of Internal Medicine, College of Public Health and Medical Sciences, Jimma University, Jimma, Ethiopia; d Jimma University Clinical Trial Unit, Jimma University, Jimma, Ethiopia; e School of Public Health, College of Medicine and Health Sciences, Hawassa University, Hawassa, Ethiopia

**Keywords:** Body weight, body mass index, HIV, antiretroviral therapy, dolutegravir, Ethiopia

## Abstract

**Background:**

The nature and burden of weight gain associated with antiretroviral treatment (ART) using a combination of Tenofovir disoproxil fumarate, lamivudine and dolutegravir (TLD) among people living with HIV (PLWH) has not been thoroughly investigated in Ethiopia. Therefore, this study aimed to evaluate changes in body weight and body mass index (BMI) in adults who initiated TLD or switched to TLD compared to those who received a non-nucleoside reverse transcriptase inhibitor (NNRTI)-based therapies.

**Methods:**

A retrospective cohort study was conducted among adult PLWH who had been receiving ART between February 2017 and October 2022 in Hawassa city administration, Sidama region. Linear mixed-effects model was used to examine BMI and body weight trends over time, while a binary logistic regression was performed to identify factors associated with *a* ≥ 10% weight gain.

**Results:**

A total of 524 adult PLWH with a median age of 35 (interquartile range: 30–41) years were included. TLD-initiated arm experienced significantly greater mean weight (8.6 kg vs. 4.95 kg, p < 0.0001) and BMI (3.11 kg/m^2^ vs. 1.84 kg/m^2^, p < 0.0001) increase than the NNRTI-based arm at two years. However, the switched arm showed no significant difference in weight (5.6 kg) and BMI (2.13 kg/m^2^) compared to the NNRTI-based arm (*p* > 0.05). There was a significant interaction effect between ART regimens and time in predicting weight and BMI gain (*p* < 0.01). Initiating ART with TLD had higher odds of ≥10% body weight gain at two years (adjusted odds ratio [AOR]: 1.9; 95% CI: 1.19–3.04). Other baseline factors such as age ≥40 years (AOR: 2.02; 95% CI: 1.35–3.02), weight <50kg (AOR: 3.0; 95% CI: 1.86–4.84), advanced disease stages (AOR: 1.78; 95% CI: 1.1–2.86) and ambulatory-bedridden functional status (AOR: 2.0; 95% CI: 1.05–3.8) were also associated with ≥10% weight gain.

**Conclusion:**

Initiating ART with TLD was significantly associated with greater weight and BMI gain than the NNRTI-based regimens. Therefore, the cardio-metabolic implications of weight gain after the TLD initiation in this population should be monitored and thoroughly investigated.

## KEY MESSAGES

- A significant interaction effect was observed between ART regimens and time in predicting weight and BMI gain.
- Starting ART with TLD was associated with a significant weight gain and treatment-emergent overweight/obesity than the NNRTI-based therapies.
- ART initiating with TLD had 1.9 times higher odds of >10% body weight gain than the NNRTI-based therapies

## Introduction

Weight gain after starting antiretroviral treatment (ART) is common, specifically in people with very low CD4+ count or lower pre-ART body mass index (BMI) [1]. Weight gain following ART initiation is frequently interpreted as evidence of treatment success and nutritional rehabilitation and it is associated with increased survival [2] and immunologic reconstitution [1]. A substantial weight gain which is associated with ART, on the other hand, may increase the risk of comorbidity with metabolic and cardiovascular diseases [3]. In particular, obesity is a significant risk factor for cardiovascular disease [4], type 2 diabetes [5], sleep apnea, psychiatric problems, several musculoskeletal diseases, and some malignancies [6].

In terms of ART regimens, integrase strand transfer inhibitors (INSTIs) are now recommended as preferred first- and second-line ART modalities for people living with HIV (PLWH) [7], due to their strong barrier to resistance and higher tolerability [7,8]. However, recent studies showed that excess weight gain or clinical obesity was more frequent among individuals taking INSTI-based ART classes, particularly dolutegravir (DTG) compared to individuals in other antiretroviral classes [9]. In addition, factors like African descent, female gender, and high pre-treatment HIV viral load or low baseline CD4+ count were also associated with moderate to excess weight gain [10]. Ethiopia changed its guideline to use INSTI-based combinations as preferred first- and second-line ART regimens in 2018 [11] following the World Health Organization (WHO) recommendation [12]. However, only limited data are available concerning the effect of INSTIs-based ART regimens on body weight and BMI in the Ethiopian population. As a result, we investigated whether initiating or switching to INSTI-based first-line therapy is associated with weight gain compared to non-nucleoside reverse transcriptase inhibitor (NNRTI)-based first-line therapies among adult PLWH after 24 months of treatment follow-up.

## Materials and methods

A retrospective cohort study was conducted among adult PLWH who had been receiving antiretroviral medications between February 20, 2017 and October 30, 2022. We collected data from seven public and private health facilities that provide ART service for PLWH in Hawassa city administration, Sidama regional state. These health facilities were Adare General Hospital, Alatyon Hospital, Hawassa University Comprehensive Specialized Hospital (HUCSH), Hawela-Tulla Hospital, Bushulo Health Center, Millennium Health Center, and Tulla Health Center. The medical records of the study participants were accessed from November 01 to December 15, 2022.

### Inclusion criteria

All PLWH who were at least 18 years of age, had been on first-line ART for at least two years and had started ART for the first time after Ethiopia implemented the WHO test-and-treat strategy (February 2017) [12,13] were included. Only adult PLWH who had suppressed HIV-1ribonuclic acid or (HIV-1 RNA <1000 copies/mL after twelve months of ART initiation) were eligible for the study. Regarding ART regimens, the following treatment arms were considered for weight and BMI analysis: [1]. Patients who had started treatment with Tenofovir disoproxil fumarate (TDF) plus lamivudine (3TC) and DTG (TLD) at the baseline and stayed on the same regimen for 24 months and had at least five of the consecutive weight data starting from the baseline (TLD arm) [2]. Those who started treatment with two nucleos(t)ide reverse transcriptase inhibitors (N(t)RTIs) plus either efavirenz (EFV) or Nevirapine (NVP) at the baseline and then switched to the TLD regimen within 24 months, and had received the initial EFV/NVP-based regimens for at least six months prior to switching and/or at least one post-switch weight data during the 24-month follow-up (switch arm) [3]. While the comparison group included individuals who initiated treatment with two N(t)RTIs plus either EFV or NVP at the baseline, remained on the same regimens and had at least five weight data from the baseline throughout the 24-month follow-up (NNRTI-based arm).

### Exclusion criteria

Adults who initiated ART before the start of the test-and-treat strategy, which means those who initiated ART before February 2017, those with poor adherence to ART therapy, those transferred in from other health facilities, those who switched to TLD before six months of therapy, and women who were pregnant at any point during 24-month follow up were all excluded from the study. In addition, participants whose follow-up records did not include the baseline and follow-up weight data for at least 24 months and those who were lost to follow-up or died before 24 months were also excluded. For more information, the inclusion and exclusion of the study participants are indicated in the flow chart (Figure S1).

### Data collection tools and procedures

A standard checklist was used to collect the information from participants’ medical records. This checklist was developed using the ART initiation and follow-up form used in the ART clinics. The following data were collected using this checklist: socio-demographic factors such as age, gender, marital status, religion, residence, occupation, and educational levels; clinical information such as duration since HIV diagnosis, duration of ART experience, baseline WHO disease stage, functional status, history of tuberculosis (TB) infection, type of TB infection, presence of opportunistic infections (OIs) other than TB, treatment with anti-TB, use of TB prophylaxis treatment (TPT), opportunistic infections prophylaxis treatment (OPT), comorbid health problems, baseline ART regimens, modification on ART regimens (switching); anthropometric measurements such as weight over 24 months and height values of the individuals. All compulsory data were collected by health professionals with the critical review of each participant’s medical chart under the supervision of the principal investigator.

### Outcome assessment

Percentage weight gain from baseline was calculated for each individual and adapted to (weights at x-month minus weight at baseline/weight at baseline) x100 [14]. In addition, moderate weight gain was defined as a 5 to <10% increase in weight from baseline, whereas excess weight gain was defined as *a* ≥ 10% increase in body weight or BMI by ≥1kilogram/square meter (kg/m^2^) at 12 or 24 months of treatment follow up [9,10,15–17]. For individuals who had recorded height values, BMI was calculated as weight (kg) divided by the square of height in meters (m^2^) and then divided into four categories as follows: underweight (<18.5 kg/m^2^), normal weight (18.5–24.9 kg/m^2^), overweight (25.0–29.9 kg/m^2^), and obese (≥30kg/m^2^) [18,19].

### Ethical approval

The Institutional Review Board at the College of Natural and Computational Sciences (CNS-IRB) at Addis Ababa University granted ethical approval for the study (approval No: IRB/07/14/2022) and waived the requirement to obtain informed consent as this study used de-identified secondary data from medical records. A letter of approval received from Addis Ababa University was submitted to the Hawassa city administration’s health department office for evaluation and writing of support letters for each study health facility. Then, an official letter of support, received from the Hawassa city health department office, was submitted to the Medical director’s office of the seven health facilities to obtain an official approval for data collection. In addition, the study complied with the standards of the Helsinki Declaration and its subsequent amendments. The confidentiality of study participants’ personal information was highly preserved.

### Statistical analysis

Means with 95% confidence intervals (CI) or medians with interquartile ranges (IQRs) were utilized as summary measures for continuous variables. Baseline demographic and clinical characteristics across the ART treatment arms were evaluated using the Pearson χ2 test, student t-test, or one-way ANOVA as appropriate. The effect of treatment with a DTG-based first-line regimen on weight/BMI over time was analyzed using linear mixed-effects model with autoregressive error structure of order 1 treating each participant as a random effect and adjusting for age, sex, TB coinfection and time. The main outcome of interest in the present study was weight gain of ≥10% over the 24-month ART follow-up. The effect of DTG-based first-line regimens on ≥10% body weight gain was investigated using multivariable binary logistic regression with adjusting for the effects of age at ART commencement, sex, duration since HIV diagnosis, baseline weight, TB infection, baseline WHO clinical stage, baseline functional status, TPT, and OPT. All covariates with a p-value of < 0.25 in bivariate analysis were included in the multivariable analysis except for sex. An alpha threshold of 5% was set for statistical significance. Stata version 17.0 (StataCorp LLC, College Station, Texas) was used for the linear mixed effects analysis, while other analyses were done using IBM SPSS Statistics version 26.0 (IBM Corp., Armonk, NY, USA).

## Results

### Baseline characteristics of the study participants

A total of 6,300 adults were receiving ART at seven health facilities during the time of data collection. Of these, 524 adults met the inclusion criteria for body weight analysis. Also, only 482 (92%) of the 524 adults had recorded height values and BMI was calculated for them. The study participants had a median age of 35 (IQR: 30–41) years at baseline and 56.7% (297/524) were females (Table 1).

**Table 1.** Baseline socio-demographic features of the study population.

Variable					ART regiments at baseline	P-value
	Total	NNRTI-based arm	TLD arm			
	*n* = 524	*n* = 367	*n* = 157			
Gender				
Female	297(56.7)	213(58.0)	84(53.5)	0.337
Male	227(43.3)	154(42.0)	73(46.5)	
Age in years, median(IQR)	35(30–41)	35(29–42)	35(30–40)	0.617
Marital status	*(n = 485)*	*(n = 350)*	*(n = 135)*	
Single	76(15.7)	55(15.7)	21(15.5)	0.88
Married	262(54)	189(54)	73(54.1)	
Divorced	83(17.1)	62(17.7)	21(15.5)	
Widow/widower	64(13.2)	44(12.6)	20(14.8)	
Residence	*(n = 509)*	*(n = 359)*	*(n = 150)*	
Rural	78(15.3)	51(14.2)	27(18.0)	0.279
Urban	431(84.7)	308(85.8)	123(82.0)	
Religion	*(n = 493)*	*(n = 357)*	*(n = 136)*	
Orthodox	185(37.5)	132(37.0)	53(39.0)	0.899
Protestant	256(51.9)	186(52.1)	70(51.5)	
Muslim	44(8.9)	34(9.5)	10(7.3)	
Catholic	5(1.0)	3(0.8)	2(1.5)	
Others	3(0.6)	2(0.6)	1(0.7)	
Occupation	*(n = 321)*	*(n = 228)*	*(n = 93)*	
Employed*	202(62.9)	140(61.4)	62(66.7)	0.087
No job	18(5.6)	14(6.1)	4(4.3)	
Daily laborers	16(5.0)	9(3.9)	7(7.5)	
Housewife	68(21.2)	54(23.7)	14(15.0)	
Farmers	10(3.1)	7(3.1)	3(3.2)	
Students	6(1.9)	4(1.7)	2(2.1)	
Commercial sex workers	1(0.3)	0(0.0)	1(1.1)	
Education	*(n = 489)*	*(n = 351)*	*(n = 138)*	
Unable to read and write	51(10.4)	39(11.1)	12(8.7)	0.061
Primary level (grade 1–8)	143(29.2)	112(31.9)	31(22.5)	
Secondary (grade 9–12)	185(37.8)	130(37.0)	55(39.8)	
College and above	110(22.5)	70(19.9)	40(29.0)	

Values presented in number (%) unless otherwise indicated; *, (include government, private and self-employed); IQR: interquartile range; ART: antiretroviral therapy; TLD: Tenofovir disoproxil fumarate plus lamivudine and dolutegravir; NNRTI-based: nucleos(t)ide reverse transcriptase inhibitors plus non-nucleoside reverse transcriptase inhibitor.

### Baseline anthropometric and clinical characteristics of the study population

Ninety-seven (18.5%) PLWH had a history of tuberculosis infection, 72 (74.2%) of them had pulmonary tuberculosis, and 25 (25.8%) had extra-pulmonary tuberculosis. Likewise, 47(8.9%) subjects had opportunistic infections other than tuberculosis (Herpes zoster (*n* = 29), pneumocystis pneumonia (*n* = 4), toxoplasmosis (*n* = 4), and candidiasis infection (*n* = 10)). Of the 524 adults, 70% (367/524) initiated ART with the NNRTI-based regimens, while 30% (157/524) initiated the TLD regimen. Of those who initiated the NNRTI-based regimens, 98.4% received TDF + 3TC + EFV, while 1.1%, 0.3%, and 0.3% received Zidovudine (ZDV) + 3TC + EFV, TDF + 3TC + NVP and abacavir (ABC)+3TC + EFV, respectively (Table 2). One hundred seventy-nine (34.1%) participants continued receiving the NNRTI-based regimens for 24 months; while 35.9% (188/524) adults were switched from the NNRTI-based regimens to TLD regimen before 24 months of treatment as per the WHO guidelines. Forty-two out of 524 (8.0%) adults had no documented height values to calculate the BMI and only 482 adults were considered for the BMI analysis. Of the 482 participants, 69.5% (335/482) initiated ART with the NNRTI-based regimens, while the remaining 30.5% (147/482) initiated the TLD regimen. About 33.8% (163/482) participants continued receiving the NNRTI-based regimens; while 35.7% (172/482) were switched from NNRTI-based regimens to the TLD regimen as per the WHO directions.

**Table 2.** Baseline anthropometric and clinical features of the study population.

Parameter					ART regiments at baseline	
	Total *n* = 524	NNRTI-based arm *n* = 367	TLD arm *n* = 157	p value		
Duration since HIV diagnosis(months)*	48(37–56)	53(47–60)	34(28–37)	***<0.0001***
Duration since ART start (months) *	46(36–54.7)	51(45–58)	34(28–37)	***<0.0001***
Weight (kg)#	58.6(57.4–59.6)	58.8(57.5–60)	58.4(56.4–60.3)	0.74
BMI(kg/m^2^)#	21.5(21.1–21.8)	21.7(21.1–22.1)	21.1(20.5–21.7)	0.161
BMI (kg/m^2^)	*n* = 482	*n* = 335	*n* = 147	
Underweight (<18 kg/m^2^)	109(22.6)	72(21.5)	37(25.2)	0.289
Normal weight (18–24.9 kg/m^2^)	279(57.9)	193(57.6)	86(58.5)	
Overweight (≥25 kg/m^2^)	82(17.0)	59(17.6)	23(15.6)	
Obese	12(2.5)	11(3.3)	1(0.7)	
WHO clinical stage				
Stage-I	215(41.0)	165(45.0)	50(31.8)	***0.022***
Stage-II	132(25.2)	88(24.0)	44(28.0)	
Stage-III	119(22.7)	73(19.9)	46(29.3)	
Stage-IV	58(11.1)	41(11.2)	17(10.8)	
Functional status				
Working	447(85.3)	315(85.8)	132(84.1)	0.776
Ambulatory	69(13.2)	46(12.5)	23(14.6)	
Bedridden	8(1.5)	6(1.6)	2(1.3)	
History of Tuberculosis infection				
No	427(81.5)	303(82.6)	124(79.0)	0.334
Yes	97(18.5)	64(17.4)	33(21.0)	
OIs other than Tuberculosis				
No	477(91.0)	327(89.1)	150(95.5)	***0.018***
Yes	47(9.0)	40(10.9)	7(4.5)	
Receipt of TPT				
No	139(26.5)	93(25.3)	46(29.3)	0.314
Yes	385(73.5)	274(74.5)	111(40.7)	
Other known chronic diseases				
No	520(99.2)	364(99.2)	156(99.4)	0.81
Yes	4(0.8)	3(0.8)	1(0.6)	
ART regimens				
ZLE(ZDV + 3TC + EFV)	4(0.8)	4(1.1)	0(0.0)	
TLE(TDF + 3TC + EFV)	361(68.9)	361(98.4)	0(0.0)	
TLN(TDF + 3TC + NVP)	1(0.2)	1(0.3)	0(0.0)	
TLD(TDF + 3TC + DTG)	157(30.0)	0(0.0)	157(100)	
ALE(ABC + 3TC + EFV)	1(0.2)	1(0.3)	0(0.0)	–
Receipt of OPT				
No	139(26.5)	109(29.7)	30(19.1)	***0.012***
Yes	385(73.5)	258(70.3)	127(80.9)	

Values presented in number (%) unless otherwise indicated; *, values in median and interquartile range; #, values in means and 95% confidence interval; ABC: Abacavir; CI: confidence interval; DTG: dolutegravir; EFV: efavirenz; 3TC: lamivudine; OIs: opportunistic infections; OPT: opportunistic infections prophylaxis treatment (cotrimoxazole); TPT: Tuberculosis prophylaxis treatment (isoniazid (INH) or isoniazid-rifapentine (3HP)); Kg: kilogram; m: meter; NNRTI-based: non-nucleoside reverse transcriptase inhibitor plus backbone nucleos(t)ide reverse transcriptase inhibitors; TLD: Tenofovir disoproxil fumarate plus lamivudine and dolutegravir; ZDV: zidovudine.

#### Body weight and BMI increase after ART initiation

**Figure 1.**
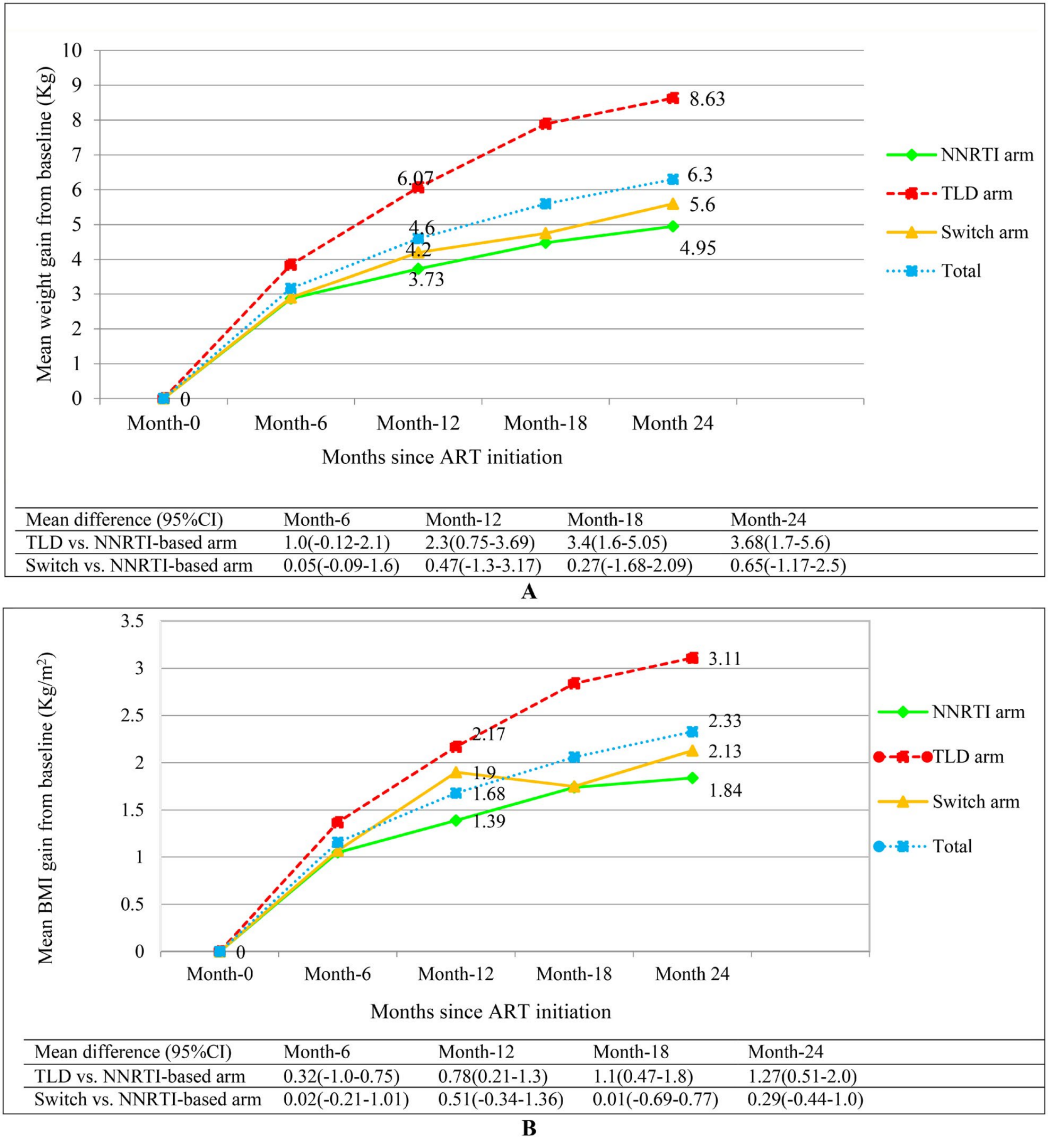
Body weight and BMI increase across treatment arms during ART follow-up (weight increase: A and BMI increase: B). Abbreviations: BMI: body mass index; Kg: kilogram; m^2^: meter square; NNRTI: non-nucleoside reverse transcriptase inhibitor plus backbone nucleos(t)ide reverse transcriptase inhibitors; TLD: Tenofovir disoproxil fumarate plus lamivudine and dolutegravir; (p-value for difference in weight gain between the TLD initiated vs. NNRTI-based arm: 0.001 at month-12; <0.0001 at month-18 and month-24); (p-value for difference in BMI gain between the TLD initiated vs. NNRTI-based arm: 0.003 at month-12; <0.0001 at month-18 and month-24).

Overall, body weight increased by 4.6 kg (95% CI: 4.06–5.15 kg) at 12 months, and 6.3 kg (95% CI: 5.65–6.94 kg) at 24 months. At 24 months, participants who started TLD gained a mean of 8.6 kg (95% CI: 7.35–9.92 kg) compared to 4.95 kg (95% CI: 3.87–6.02 kg) gain in the NNRTI-based arm (*p* < 0.0001). Weight gain in the switched arm (5.6 kg; 95% CI: 4.65–6.61 kg) was greater than weight gain in the NNRTI-based arm, but the difference was not statistically significant (*p* > 0.05) (Figure 1A).

Similarly, the BMI increased by 1.7 kg/m^2^ (95% CI: 1.47–1.89 kg/m^2^) at month 12, and 2.3 kg/m^2^ (95% CI: 2.08–2.58 kg/m^2^) at month 24. Participants who initiated the TLD regimen gained a mean of 3.11 kg/m^2^ (95%CI: 2.62–3.60 kg/m^2^) compared to 1.84 kg/m^2^ (95% CI: 1 .44–2.25 kg/m^2^) gain in the NNRTI-based arm at 24 months, (*p* < 0.0001) (Figure 1B).

**Figure 2.**
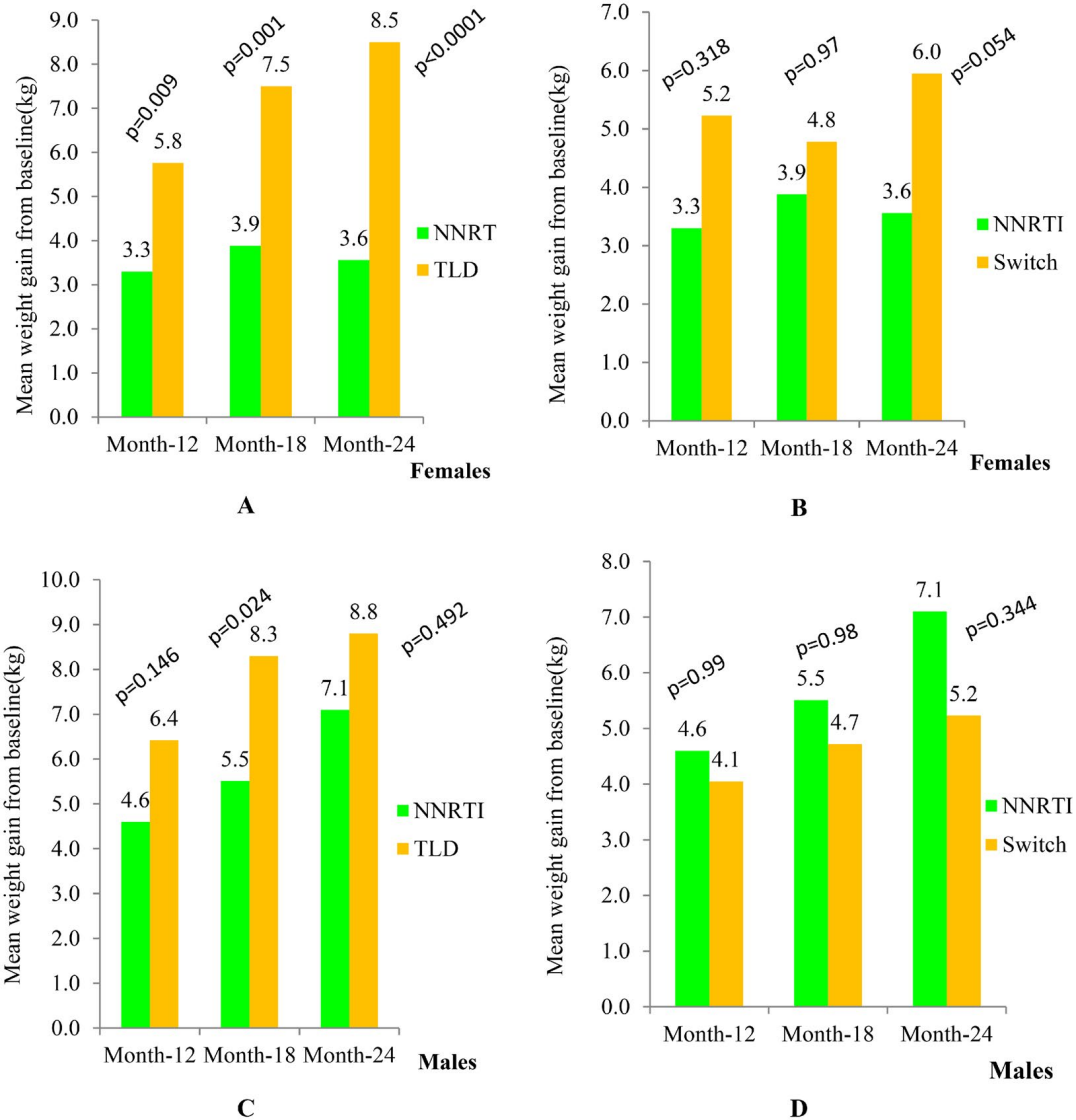
Body weight increase across treatment arms after ART initiation. Abbreviations: Kg: kilogram; NNRTI: non-nucleoside reverse transcriptase inhibitor plus backbone nucleos(t)ide reverse transcriptase inhibitor; TLD: Tenofovir disoproxil fumarate plus lamivudine and dolutegravir; switch, transitioned from NNRTI-based regimen to TLD.

Females who started the TLD regimen gained significantly more weight than females in the NNRTI-based arm (5.8 kg vs. 3.3 kg, *p* = 0.009) at 12 months, and (8.6 kg vs. 3.6 kg, *p* < 0.0001) at 24 months, respectively (Figure 2A). However, females in the switch arm gained insignificantly greater weight than females in the NNRTI-based arm (Figure 2B). In addition, males who started the TLD regimen gained more weight at 18 months compared to males in the NNRTI-based arm (8.3 kg vs.5.5 kg, *p* = 0.024), respectively (Figure 2C). However, weight gain among males in the switch arm was not significantly different from those males in the NNRTI-based arm (Figure 2D). Moreover, the BMI gain also revealed similar trends like body weight (Figure S2).

Regarding intra-regimen comparison, only males in the NNRTI-based arm gained significantly greater weight than females in the NNRTI-based arm at month 18 (5.5 kg vs.3.9 kg, *p* = 0.047), and at month 24 (7.1 kg vs. 3.6 kg, *p* = 0.001).

In the linear mixed-effects model, the mean weight of individuals who started ART with a TLD regimen was 0.23 kg lower than that of those who started ART with an NNRTI-based regimen (β = −0.23; 95% CI: −2.43–1.96; *p* = 0.84). After controlling for time, age, TB co-infection, and gender, the mean weight of the switched arm was 1.82 kg greater than the NNRTI-based arm (β = 1.82; 95% CI: 0.35–3.30; *p* = 0.015). Individuals infected with TB at the start of ART had a 3.77 kg lower mean weight than those who were not infected with TB (β =-3.77; 95% CI: −6.31 to −1.24; *p* = 0.004). In addition, the mean weight of male individuals was 5.75 kg greater than that of female participants (β = 5.75; 95% CI: 3.73–7.77, *p* < 0.0001). Time was also found to be a significant predictor of weight gain. The weight increased by 1.44 kg as the treatment time increased by six months (β = 1.44; 95% CI: 1.23–1.66, *p* < 0.0001). Statistically significant interaction effects between time and ART regimens have been observed, with treatment starting with a TLD regimen having 0.71 kg higher weight (β = 0.71; 95% CI: 0.34–1.07; *p* < 0.0001) and 0.244 kg/m^2^ higher BMI (β = 0.244; 95% CI: 0.10–0.39; *p* = 0.001) than the NNRTI-based regimens (Table S1).

#### Incidence of excess weight and BMI gain at 24 months of ART follow up

In total, 262 (50%) of PLWH had gained ≥10% weight after 24 months of ART, of these, 50.2% of them were females. The proportion of PLWH who gained ≥10% weight was 44.1%, 59.2%, and 47.9% for the NNRTI-based arm, TLD arm and the switched arm, respectively. A weight gain ≥10% occurred more frequently in the TLD-initiated arm (59.2%) than in the NNRTI-based arm (44.1%), *p* = 0.01 (Table S2). In addition, 40.4%, 61.9%, and 51% of females experienced ≥10% weight gain in the NNRTI-based arm, TLD arm, and the switched arm compared to 50%, 56.2% and 44% of males, respectively.

**Figure 3.**
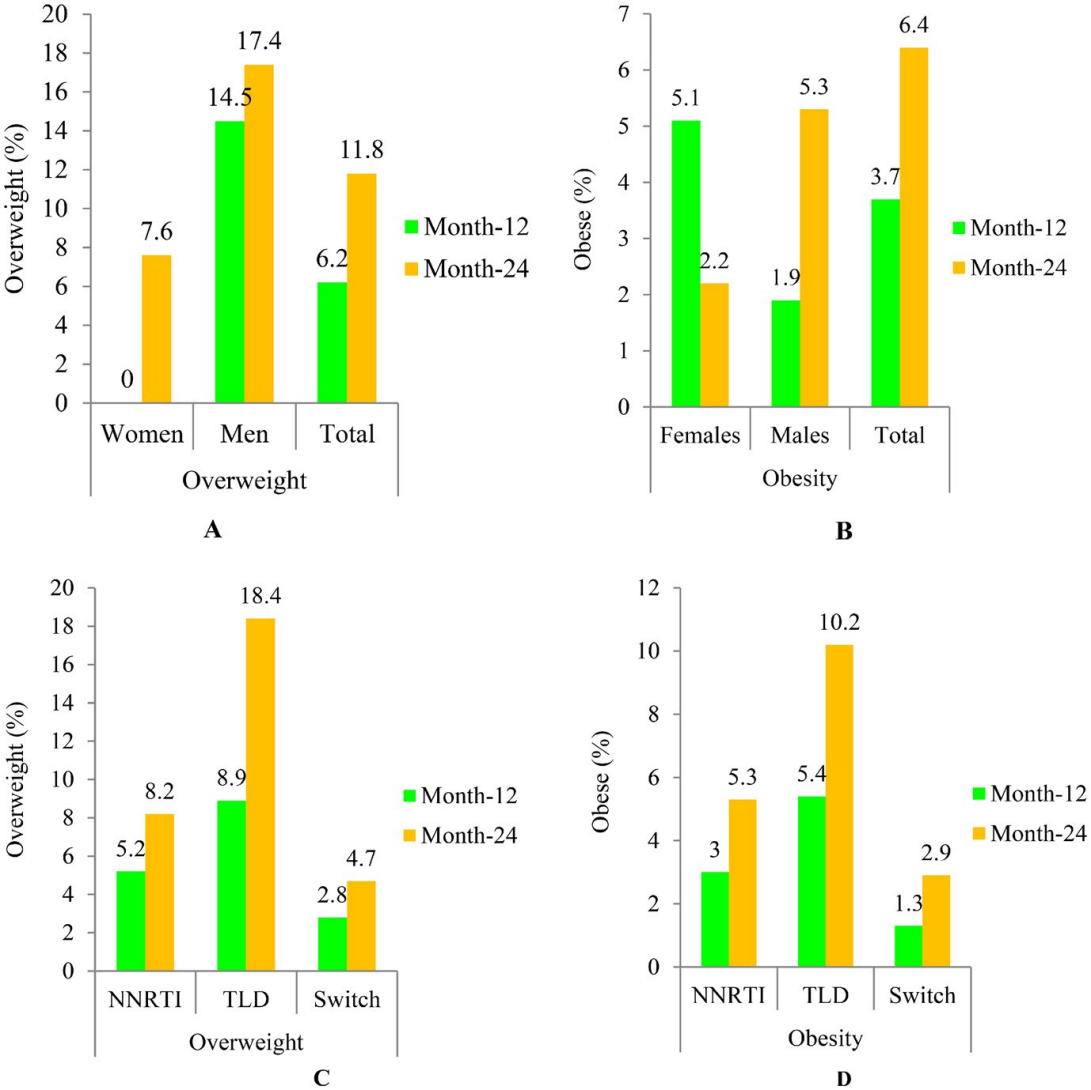
The proportion of newly emerged overweight/obesity after ART initiation (gender-based: A and B; and regimens-based: C and D). Abbreviations: Overweight (BMI = 25–29.9kg/m^2^); Obesity(BMI ≥30kg/m^2^); NNRTI based arm, non-nucleoside reverse transcriptase inhibitor plus backbone nucleos(t)ide reverse transcriptase inhibitors; TLD: Tenofovir disoproxil fumarate plus lamivudine and dolutegravir; switch arm, transitioned from NNRTI-based regimens to TLD.

The rate of participants who gained BMI ≥1kg/m^2^ was 66.2% (319/482);  of them, 56.4% (180/319) were females (Table S3). About 18.2% of participants became newly overweight to obese (BMI ≥25 kg/m^2^), with 11.8% overweight and 6.4% obese. In addition, 17.4% of males became overweight (Figure 3A), while 5.3% of males and 2.2% of females became newly obese (Figure 3B). Treatment-emergent overweight and obesity were greater in the TLD-started arm compared to the NNRTI-based arm (Figure 3C,D). A BMI gain of ≥1kg/m^2^ was considerably higher in individuals with a baseline BMI of <25kg/m^2^ than those with a baseline BMI of ≥25 kg/m^2^ (83.4% vs. 16.6%; *p* = 0.037), respectively (Table S4). Moreover, 18.4% and 10.2% of BMI-normal adults who started the TLD regimen became overweight and obese, respectively, compared to 11.8% and 5.3% of those who received the NNRTI-based therapies.

### Factors associated with ≥10% weight gain after 24 months of ART follow up

To determine factors associated with body weight gain, we used ≥10% weight gain as an outcome variable after 24 months of treatment follow-up. Treatment initiation with TLD regimen resulted in higher odds of ≥10% weight gain than treatment initiation with NNRTI-based regimens. Adults who initiated the TLD regimen had 1.9 times greater odds to gain ≥10% of their initial weight than those who started the NNRTI-based regimens (adjusted odds ratio [AOR]:  1.9; 95% CI: 1.19–3.04) (Table 3).

**Table 3.** Factors associated with ≥10% of weight gain at 24 months after ART initiation.

Variable category	Weight gain after ART initiation				
	Total	<10%	≥10%	COR	AOR 95%CI
	524(%)	262 (%)	262 (%)	95%CI	
Gender					
Females	297(56.7)	148(56.5)	149(56.9)	1.02(0.72–1.4)	1.06(0.72–1.58) 1.00
Male	227(43.3)	114(43.5)	113(43.1)	1.00	
Age					
<40 years old	346(66)	190(72.5)	156(59.5)	1.00	1.00 2.02(1.35–3.02)**
≥40 years old	178(34)	72(27.5)	106(40.5)	1.79(1.24–2.59)**	
Baseline body weight					
<50 kg	124(23.7)	35(13.4)	89(34.0)	3.34(2.1–5.2)**	3.0(1.86–4.84)** 1.00
≥50 kg	400(76.3)	227(86.6)	173(66.0)	1.00	
ART regimen arms					
NNRTI-based arm	179(34.2)	100(38.2)	79(30.2)	1.00	1.00 1.9(1.19–3.04)** 1.2(0.76–1.88)
TLD arm	157(30.0)	64(24.4)	93(35.5)	1.84(1.19–2.84)**	
Switch arm	188(35.9)	98(37.4)	90(34.4)	1.16(0.77–1.75)	
History of tuberculosis infection					
No	427(81.5)	232(88.6)	195(74.4)	1.00	1.00 1.36(0.62–2.96)
Yes	97(18.5)	30(11.5)	67(25.6)	2.66(1.66–4.25)**	
Baseline WHO clinical stage					
I and II	347(66.2)	204(77.9)	143(54.6)	1.00	1.00 1.78(1.1–2.86)*
III and IV	177(33.8)	58(22.1)	119(45.4)	2.93(2.0–4.28)**	
Baseline functional status					
Working	447(85.3)	244(93.1)	203(77.5)	1.00	1.00 2.0(1.05–3.8)*
Ambulatory and bedridden	77(14.7)	18(6.9)	59(22.5)	3.94(2.25–6.89)**	
TPT received					
No	138(26.3)	54(20.6)	84(32.1)	1.82(1.22–2.7)**	1.0(0.54–1.9) 1.00
Yes	386(73.7)	208(79.4)	178(67.9)	1.00	
OPT received					
No	139(26.5)	88(33.6)	51(19.5)	1.00	1.00 1.35(0.87–2.12)
Yes	385(73.5)	174(66.4)	211(80.5)	2.09(1.4–3.12)**	

AOR: adjusted odds ratio; CI: Confidence interval; COR: crude odds ratio; NNRTI-based: non-nucleoside reverse transcriptase inhibitor plus backbone nucleos(t)ide reverse transcriptase inhibitors; OPT: opportunistic infections prophylaxis treatment(cotrimoxazole); TPT: Tuberculosis prophylaxis treatment (isoniazid (INH) or isoniazid-rifapentine (3HP)); TLD: Tenofovir disoproxil fumarate plus lamivudine and dolutegravir; switch, transitioned from NNRTI-based regimen to TLD; WHO: World Health Organization; (overall model fit control statistics for the final model was as follows: (X^2^(df = 8) =12.841, *p* < 0.001, -2Log probability = 640.404, Nagalkerke R^2^ = 0.202, total percent predicted 67.4%, and Hosmer and Lemeshow’s test of significance, *p* = 0.117); *, *p* < 0.05; **, *p* < 0.01.

## Discussion

The study subjects who initiated ART with the TLD regimen gained significantly more weight and had greater BMI than the NNRTI-based regimens. Compared to NNRTI-based regimens, starting treatment with the TLD regimen was significantly associated with ≥10% weight gain. In addition, older age and other baseline factors such as the WHO disease stages, functional status and body weight were associated with ≥10% weight gain.

In this study, participants who started ART with the TLD regimen gained significantly more weight than those who started the NNRTI-based regimens after 12- and 24 months. This finding is consistent with several clinical trials and observational studies that examined the same problem in other populations, such as a recent 48-week open-label randomized trial in South Africa [20], an 18-month cohort study in South-eastern US [9], a 2 and 5-year cohort study in the Northern US and Canada [9], a meta-analysis of eight clinical trials [10], and a 48-week open-label, multicentre, randomized study in Cameroon [21]. In support, the use of INSTI medications has been associated with more weight gain than PIs or NNRTIs; however, elvitegravir/cobicistat was associated with lesser weight gain than DTG and bictegravir. Among NNRTIs, rilpivirine was associated with higher weight gain than efavirenz. Among N(t)RTIs backbone, Tenofovir alafenamide (TAF) is also associated with higher weight gain than TDF, ABC, or ZDV [10]. In addition, weight gain associated with INSTI drugs has also been reported in low- and middle-income countries [21] and also resource-rich settings [10]. The exact mechanism of drug-induced weight gain by INSTIs is unknown, but some clinical studies suggest that INSTIs may impair appetite regulation in the central nervous system (CNS), particularly melanocortin-4 receptor (MC4R), which is a key receptor in the CNS that regulates caloric intake through modulating leptin signaling and is involving in energy homeostasis and appetite regulation [22]. Supportively, studies *in vitro* animal models have shown that MC4R knockout mice suffer from severe obesity [23,24] and the MC4R gene is shared by both mice and humans.

PLWH who initiated treatment with the TLD regimen gained an average BMI of 3.1 kg/m^2^, while the switch arm also gained 2.1 kg/m^2^ at 24 months of ART follow-up. This may increase the risk of metabolic and cardiovascular disease with long-term treatment in this population. For instance, a study report revealed that a 1 kg/m^2^ increase in BMI after starting ART could have a 12% increased risk of developing diabetes and an 18–20% increased risk of developing cardiovascular disease, irrespective of the pre-ART BMI [4]. In addition, the study findings from Veterans Affairs showed that PLWH had a 14% increased risk of developing diabetes mellitus when their weight increased by 5% compared to veterans without HIV infection [5].

Several studies reported a greater weight gain after subjects switched from protease inhibitors (PIs)- or NNRTI-based regimens to INSTIs-based regimens compared to those who received the NNRTI-based regimens; however, the difference was not statistically significant [25–28]. This is consistent with the findings of the present study, which indicate greater weight gain in the switch arm compared to the NNRTI-based arm after 24 months of ART. Contrary to our findings, several studies showed that virologically suppressed people who received the NNRTI-based therapies and then switched to the INSTI-based drugs, particularly a combination of DTG, gained significantly more weight than those who continued the NNRTI-based regimens [10,27,29,30]. Treatment experience with non-INSTI-based regimens before switching, length of time with INSTIs-based drugs after switching, and practice of switching prior to INSTIs may have contributed to the differences.

In this study, ART initiation with TLD regimen predicted significantly greater weight and BMI gain over time, and ART initiation with TLD resulted in 1.9 times higher odds of ≥10% weight gain than the NNRTI-based regimens. Similarly, a meta-analysis of eight clinical trials conducted by Sax et al. [10] and other observational studies [9,27,31] have shown a significant association between weight gain and INSTI-based regimens in general and the DTG-based regimens in particular, with greater weight gain compared to the NNRTI- or PI-based therapies. The finding of this study indicated that subjects with ambulatory and bedridden functional status had 2.0 times higher odds to gain ≥10% of their initial weight than those with working functional status. This finding is consistent with other studies conducted elsewhere [32–34]. In addition, the present study indicated that people with advanced WHO clinical stages (stages III and IV) during ART initiation had 1.78 times higher odds to gain ≥10% of their initial weight than those with mild clinical stages (stages I and II). The finding is in line with the studies conducted elsewhere [10,35,36], and this may reflect the severity of HIV disease prior to ART initiation and return to health after the start of ART particularly in individuals with more advanced clinical stages. However, other studies have reported similar effects with baseline low CD4+ count [10,31,37,38] and baseline high HIV-1 RNA copies [10,36,39]. The inclusion criteria in our study was targeted at the WHO’s test-and-treat strategy [12,13], which focuses only on a positive laboratory diagnosis of HIV result for ART initiation, regardless of CD4+ cells level or the WHO clinical stages of the individuals. Moreover, those individuals who weighed less than 50 kg at baseline and were ≥ 40 years old had 3.0 times and 2.0 times greater odds to gain ≥10% of their initial weight, respectively than their counterparts. Similarly, a study report from Tanzania [40] and other studies showed a significant association between baseline body weight and older age with excess weight gain [39,41]. Large-scale studies also revealed people who were underweight at the baseline of ART and had evidence of more advanced untreated HIV infection gained ≥10% of their body weight during ART follow-up [42].

Moreover, linear mixed-effects model analysis revealed that individuals infected with TB at the start of ART had a 3.77 kg lower mean weight than those who were not infected with TB. Similarly, the study indicated that people with advanced disease stages may have poor weight gain because of OIs, particularly TB, or increased energy expenditure from higher metabolic demands [35].

Furthermore, this study showed a significant difference in weight and BMI increase across treatment groups within female participants, however; females did not show a significant association with ≥10% weight gain compared to males. Consistent with our findings, a recent large multi-cohort study showed a non-significant association between gender and weight gain [43]. In contrast to the present study, previous studies reported an association between weight gain and female sex, particularly among those who initiated treatment with INSTIs-based regimens [10,31,39,41]. This could be attributed to the ethnic effect on weight gain, which contributes to the differences between the studies.

### Strengths and limitations of the study

As strengths, this study compared the effects of starting ART with TLD or switching to TLD therapy compared to NNRTI-based regimens on weight and BMI increase and potential predictors of excess weight gain in seven ART-providing healthcare facilities. In addition, we included all adults who started first-line ART after Ethiopia adopted the WHO test-and-treat strategy [12,13]. This test-and-treat strategy only focuses on a positive HIV diagnosis for ART initiation, regardless of the CD4+ count, WHO clinical stage, or other criteria. As a result, this may be useful in limiting the participation of people with a low CD4+ count, advanced disease stages, or wasting syndrome to minimize their confounding effect on weight gaining pattern analysis.

However, this study has certain limitations associated with the design of retrospective studies. As a retrospective investigation, the results might vary from a prospectively conducted cohort study. In addition, we only included participants who had complete data on outcome variables, which could result in ascertainment bias. Confounding factors such as socioeconomic status, calorie intake, physical activity, smoking, and alcohol consumption were absent from participants’ medical records but could impact weight gain. In addition, potential factors that can contribute to weight gain such as the onset of diabetes; hypertension, dyslipidemia and use of concomitant medications were not documented in the medical charts in most health facilities and have not been evaluated.

## Conclusion

We found that DTG-based therapy (TLD) was associated with greater weight and BMI and also excess weight gain compared to NNRTI-based therapies after two years of ART initiation. A significant interaction effect was observed between ART regimens and time in predicting weight and BMI gain. In addition, older age and other baseline factors such as the WHO clinical stages, functional status, and body weight were also associated with a weight gain of ≥10%. Irrespective of the improved tolerability and efficacy of INSTI-based ART regimens, post-treatment excess weight gain however needs attention as it may increase the risk of non-HIV/AIDS comorbidities such as metabolic and cardiovascular diseases. Therefore, the cardio-metabolic implications of weight gain after the initiation of DTG-based regimens in this population should be monitored and thoroughly investigated.

## Supplementary Material

Supplemental Material

## Data Availability

The data are not publicly available due to privacy or ethical restrictions and is available upon reasonable request to the corresponding author with the authorization of Hawassa city administration, health department office, and ethics committee.
