# Supplementary material for: Effect of dolutegravir-based first-line antiretroviral therapy on weight and body mass index among adult people living with HIV on follow up at health facilities in Hawassa city administration, Southern Ethiopia: a retrospective cohort study
Source: Ann Med. 2023 Aug 2;55(2):2242250. doi: 10.1080/07853890.2023.2242250 (PMC10399476; doi:10.1080/07853890.2023.2242250)
Supplement: Supplemental Material [file IANN_A_2242250_SM5596.docx]

Number of PLWH on ART at Hawassa city administration during data collection (n= **6300)**

**Charts were excluded with reason (n=5765) because:**

- subjects started ART before the implement of test and treat strategy
- some of the subjects were children and adolescents
- subjects received ART for less than 24 months during data collection
- some of the subjects were regnant within 24 months of ART follow-up
- some were transferred in with absence of baseline and follow up data
- some of them had had poor adherence to ART treatment
- some had non-suppressed viral load level ( ≥1000 copies/ml) at the month-12
- lack of baseline and consecutive weight records
- some were receiving 2^nd^ line ART regimens and or switched to 2^nd^ the line ART regimens

Millinium Health Center= 69 charts

Eligible= 535 adult PLWH on first-line ART

Bushulo Health Center=29 charts

Tulla Health Center=9 charts

Adare general Hospital= 202 charts

Hawassa University Comprehensive Specialized Hospital =194 charts

Alatyon general Hospital= 30 charts

Hawella Tulla Hospital= 2 charts

**Excluded charts (n=11)** because the subjects switched to the TLD regimen before completing six months

Finally **24** months longitudinal data was collected from the medical charts of **524** adults who were on first**-**line ART

ART, antiretroviral therapy; TLD, Tenofovir disoproxil fumarate plus lamivudine and dolutegravir; PLWH, people living with HIV

**Figure S1.** Flow chart indicating the study subjects enrolment procedure at Hawassa city administration, Sidama region, between February 2017 and October 30, 2022.

**Table S1**. Effect of antiretroviral treatment regimens on weight and body mass index of adults adjusting for other covariates using linear mixed-effects models

|  |  | Coefficient (β) | Standard  error (SE) | 95% CI | *p-value* |
| --- | --- | --- | --- | --- | --- |
| Weight(kg) |  |  |  |  |  |
|  | Time(months) | 1.44 | 0.11 | 1.23-1.66 | *<0.0001* |
| *Main-effect* | Age (years) | 0.21 | 0.05 | 0.10-0.31 | *<0.0001* |
|  | NNRTI-based arm | ref | ref | ref |  |
|  | TLD initiated arm | -0.23 | 1.12 | -2.43-1.96 | *0.84* |
|  | Switched arm | 1.82 | 0.75 | 0.35-3.30 | *0.015* |
|  | Females | ref | ref | ref |  |
|  | Males | 5.75 | 1.03 | 3.73-7.77 | *<0.0001* |
|  | Not infected with TB | ref | ref | ref |  |
|  | Infected with TB | -3.77 | 1.29 | -6.31 to-1.24 | *0.004* |
| Weight(kg) | NNRTI-based arm | ref | ref | ref |  |
| *Interaction****:*** *treatment*  *arms on time trend* | TLD initiated arm | 0.71 | 0.19 | 0.34-1.07 | *<0.0001* |
|  | Switched arm | -0.72 | 0.23 | -1.17 to-0.27 | *0.002* |
|  | ***constant*** | 49.87 | 2.02 | 45.9-53.8 | *<0.0001* |
| BMI(kg/m^2^) |  |  |  |  |  |
|  | Time(months) | 0.53 | 0.04 | 0.45-0.62 | *<0.0001* |
|  | Age (years) | 0.09 | 0.02 | 0.05- 0.12 | *<0.0001* |
| *Main-effect* | NNRTI-based arm | ref | ref | ref |  |
|  | TLD initiated arm | -0.32 | 0.39 | -1.1- 0.43 | *0.40* |
|  | Switched arm | 0.71 | 0.28 | 0.16-1.26 | *0.012* |
|  | Females | ref | ref | ref |  |
|  | Males | -1.13 | 0.36 | -1.8 to -0.43 | *0.02* |
|  | Not infected with TB | ref | ref | ref |  |
|  | Infected with TB | -3.77 | 1.29 | -6.31 to-1.24 | *0.004* |
| BMI(kg/m^2^) | NNRTI-based arm | ref | ref | ref |  |
| *Interaction****:*** *treatment* | TLD initiated arm | 0.244 | 0.07 | 0.10-0.39 | *0.001* |
| *arms on time trend* | Switched arm | -0.25 | 0.09 | -0.43 to -0.08 | *0.003* |
|  | ***constant*** | 19.3 | 0.70 | 17.9-20.64 | *<0.0001* |

ref, reference category; BMI , body mass index*;* kg, kilogram; m, meter; NNRTI, non-nucleoside reverse transcriptase inhibitor plus backbone nucleos(t)ide reverse transcriptase inhibitors; TB, tuberculosis; TLD, Tenofovir disoproxil fumarate plus lamivudine and dolutegravir; switch, transitioned from NNRTI-based regimen to TLD

**A**

**C**

**B**

**D**

Kg, kilogram; m^2^, meter square; NNRTI based, non-nucleoside reverse transcriptase inhibitor plus backbone nucleos(t)ide reverse transcriptase inhibitors; TLD, Tenofovir disoproxil fumarate plus lamivudine and dolutegravir; switch, transitioned from NNRTI-based regimens to TLD

**Figure S2**. BMI increase across treatment arms after ART initiation

**Table S2.** Proportions of weight gain over 24 months of ART follow-up across treatment arms

| Weight gain from baseline | Total | NNRTI-based arm | TLD arm | Switch  arm | |  | TLD vs. NNRTI | Switch vs. NNRTI |
| --- | --- | --- | --- | --- | --- | --- | --- | --- |
|  | n(%) | n(%) | n(%) | n(%) | | p-value | p-value | p-value |
| ***At month 6*** | ***n=524*** | ***n=356*** | ***n=157*** | ***n=11*** | |  |  |  |
| Low (<5%) | 272(51.9) | 190(53.4) | 76(48.4) | | 6(54.5) | 0.77 | 0.49 | 0.849 |
| Moderate (5-<10%) | 116(22.1) | 78(21.9) | 35(22.3) | | 3(27.3) |  |  |  |
| Excess (≥10%) | 136(26.0) | 88(24.7) | 46(29.3) | | 2(18.2) |  |  |  |
| ***At month 12*** | ***n=524*** | ***n=315*** | ***n=157*** | | ***n=52*** |  |  |  |
| Low (<5%) | 221(42.2) | 140(44.4) | 62(39.5) | | 19(36.5) | 0.261 | 0.23 | 0.17 |
| Moderate (5-<10%) | 102(19.5) | 66(21.0) | 28(17.8) | | 8(15.5) |  |  |  |
| Excess (≥10%) | 201(38.4) | 109(34.6) | 67(42.7) | | 25(48.1) |  |  |  |
| ***At month 18*** | ***n=524*** | ***n=250*** | ***n=157*** | | ***n=188*** |  |  |  |
| Low (<5%) | 195(37.2) | 103(41.2) | 44(28.0) | | 48(41.0) | ***0.039*** | ***0.01*** | 0.898 |
| Moderate (5-<10%) | 90(17.2) | 45(18.0) | 26(16.6) | | 19(16.2) |  |  |  |
| Excess (≥10%) | 239(45.6) | 102(40.8) | 87(55.4) | | 50(42.7) |  |  |  |
| ***At month 24*** | ***n=524*** | ***n=179*** | ***n=157*** | | ***n=188*** |  |  |  |
| Low (<5%) | 183(39.4) | 72(40.2) | 40(25.5) | | 71(37.8) | ***0.039*** | ***0.01*** | 0.771 |
| Moderate (5-<10%) | 79(15.1) | 28(15.6) | 24(15.3) | | 27(14.4) |  |  |  |
| Excess (≥10%) | 262(50.0) | 79(44.1) | 93(59.2) | | 90(47.9) |  |  |  |

NNRTI-based arm, non-nucleoside reverse transcriptase inhibitor plus backbone nucleos(t)ide reverse transcriptase inhibitors; TLD, Tenofovir disoproxil fumarate plus lamivudine and dolutegravir; switch arm, transitioned from NNRTI based arm to TLD

**Table S3.** Proportion of BMI gain over 24 months of ART follow-up

| **Variables Regimens** | | | **Total**  **482(%)** | **BMI gain from baseline** | | | |
| --- | --- | --- | --- | --- | --- | --- | --- |
|  |  |  |  | **<1.0kg/m^2^** | **≥1.0 kg/m^2^** | **1.0-2.0kg/m^2^** | **>2.0 kg/m^2^** |
|  |  |  |  | **163(%)** | **319(%)** | **64(%)** | **255(%)** |
| ***Females*** |  | NNRTI-based arm | 101(20.9) | 43(26.4) | 58(18.2) | 12(18.7) | 46(18) |
|  |  | TLD arm | 77(16) | 19(11.6) | 58(18.2) | 11(17.2) | 47(18.4) |
|  |  | Switch arm | 97(20.1) | 33(20.2) | 64(20.1) | 10(15.6) | 54(21.2) |
| ***Males*** |  | NNRTI-based arm | 62(12.8) | 16(9.8) | 46(14.4) | 12(18.7) | 34(13.3) |
|  |  | TLD arm | 70(14.5) | 22(13.5) | 48(15) | 6(9.4) | 42(16.5) |
|  |  | Switch arm | 75(15.6) | 30(18.4) | 45(14.1) | 13(20.3) | 32(12.5) |
| ***Total*** |  | Females | 275(57.1) | 95(58.3) | 180(56.4) | 33(51.6) | 147(57.6) |
|  |  | Males | 207(42.9) | 68(41.7) | 139(43.6) | 31(48.4) | 108(42.3) |

BMI, body mass index; NNRTI-based arm, non-nucleoside reverse transcriptase inhibitor plus backbone nucleos(t)ide reverse transcriptase inhibitors; TLD, Tenofovir disoproxil fumarate plus lamivudine and dolutegravir; switch arm, transitioned from NNRTI-based arm to TLD

**Table S4.** Incidence of BMI gain ≥1kg/m^2^ in relation to different variables after ART initiation

| Variable Category | | | BMI gain after 24 months after ART initiation | | | |
| --- | --- | --- | --- | --- | --- | --- |
|  |  |  | Total | <1kg/m^2^ | ≥1kg/m^2^ | p-value |
|  |  |  | 482(%) | 163 (%) | 319 (%) |  |
| ***Gender*** | | |  |  |  |  |
| Females | | | 275(57.1) | 95(58.3) | 180(56.4) | 0.67 |
| Males | | | 207(42.9) | 68(41.7) | 139(43.6) |  |
| ***Age*** |  | |  |  |  |  |
|  | <40 years old | | 319(66.2) | 119(73.0) | 200(62.7) |  |
|  | ≥40 years old | | 163(33.8) | 44(27.0) | 119(37.3) | 0.024 |
| ***Baseline BMI*** | |  |  |  |  |  |
|  | | <25 kg/m^2^ | 389(80.7) | 123(75.5) | 266(83.4) |  |
|  |  | ≥25 kg/m^2^ | 93(19.3) | 40(24.5) | 53(16.6) | 0.037 |
| ***ART regimen arms*** | | |  |  |  |  |
| NNRTI based arm | | | 163(33.8) | 59(36.2) | 104(32.6) |  |
| TLD arm | | | 147(30.5) | 42(25.2) | 106(33.3) |  |
| Switch arm | | | 172(35.7) | 63(38.7) | 109(34.2) | 0.19 |
| ***History of Tuberculosis infection*** | | |  |  |  |  |
| No | | | 394(81.7) | 144(88.3) | 250(78.4) |  |
| Yes | | | 88(18.3) | 19(11.7) | 69(21.6) | 0.007 |
| ***Baseline WHO clinical stage*** | | |  |  |  |  |
| I & II | | | 319(66.2) | 131(80.4) | 188(58.9) |  |
| III & IV | | | 163(33.8) | 32(19.6) | 131(41.1) | <0.0001 |
| ***Baseline functional status*** | | |  |  |  |  |
| Working | | | 413(85.7) | 152(93.3) | 261(81.8) |  |
| Ambulatory and bedridden | | | 69(14.3) | 11(6.7) | 58(18.2) | 0.001 |
| ***TPT received*** | | |  |  |  |  |
| No | | | 121(25.1) | 34(20.9) | 87(27.3) |  |
| Yes | | | 361(74.9) | 129(79.1) | 232(72.7) | 0.12 |
| ***OPT received*** | | |  |  |  |  |
| No | | | 128(26.6) | 65(39.90 | 63(19.7) |  |
| Yes | | | 354(73.4) | 98(60.1) | 256(80.3) | <0.0001 |

BMI, body mass index; kg, kilogram; m, meter; NNRTI-based arm, non-nucleoside reverse transcriptase inhibitor plus backbone nucleos(t)ide reverse transcriptase inhibitors; OPT, opportunistic infections prophylaxis treatment(cotrimoxazole); TPT, Tuberculosis prophylaxis treatment (isoniazid (INH) or  isoniazid-rifapentine (3HP)); switch arm, transitioned from NNRTI-based arm to TLD; TLD, Tenofovir disoproxil fumarate plus lamivudine and dolutegravir; WHO, World Health Organization
